# Implication of extrinsic and intrinsic apoptotic pathways in the targeted therapy of hepatocellular carcinoma using aptamer-labeled viramidine nanoparticles

**DOI:** 10.1186/s12885-022-10201-6

**Published:** 2022-10-29

**Authors:** Ahmed A. Abd-Rabou, Hanaa H. Ahmed, Mohamed S. Kishta

**Affiliations:** 1 Hormones Department, Medical Research and Clinical Studies Institute, National Research Centre, Giza, 12622 Dokki Egypt; 2 Stem Cell Lab., Center of Excellence for Advanced Science, National Research Centre, Giza, 12622 Dokki Egypt

**Keywords:** Aptamer AS1411, Viramidine, Nanoparticles, Hepatocellular carcinoma, Apoptosis

## Abstract

Hepatocellular carcinoma (HCC) is a global health problem with regional differences in epidemiological statistics. Co-assembling the drug nanoparticles and targeting moieties could improve the therapeutic delivery of anti-cancer drugs. In this attempt, we tracked the extrinsic and intrinsic apoptotic pathways in HCC cells using viramidine (VRM)-loaded aptamer (APT) nanoparticles. In these NPs, both APT and VRM act as targeted ligands/drugs to HCC cells. The NPs were characterized using TEM, ESI–MS, FTIR, and ^1^H NMR. The results showed uniform particles with round and smooth shapes on the nano-scale. SRB-based cytotoxicity was performed and IC_50_ values were measured for HCC versus normal cells upon the proposed treatments. The flow cytometry technique was applied to determine apoptosis, then confirmed using genetic and protein analyses. In addition, nitric oxide (NO) and its enzyme (iNOS) were analyzed to examine the effect of reactive nitrogen species (RNS) on apoptosis induction. The present findings indicated that Huh-7 cells were more sensitive to APT-VRM NPs than HepG2 cells, recording the lowest IC_50_ values (11.23 ± 0.23 µM and 16.69 ± 1.12 µM), as well as the highest significant increase in the apoptotic cells (61.5% and 42%), respectively. Intriguingely, normal BHK-21 cells recorded undetectable IC_50_ values in the applied NPs, confirming their targeted delivery ability. The genetic expression and protein levels of c-FLIP, Bcl-2, and TNF-α were down-regulated, while FADD, caspase 8, caspase 3, caspase 9, and Bax were up-regulated upon treatment with APT-VRM NPs. The prepared VRM NPs labeled with APT could significantly elevate NO via activation of iNOS. In conclusion, APT-VRM NPs bioconjugate interferes with HCC cells through NO-mediated extrinsic and intrinsic apoptosis.

## Introduction

Hepatocellular carcinoma (HCC) is a worldwide disease with disparities in epidemiological statistics from one region to another region. HCC is considered the most difficult health condition in Egypt as indicated by health authorities. Last years, 5.9% individuals in Egypt were diagnosed as chronic liver disease patients with a remarkable increase in the percentage of men (around 87.6%). Arround 66.8% of the last percentage was predominant age group (40–59 years), indicating a move toward younger men. In addition, the number of HCC patients in Egypt is increased by twofold over last 10 years [1]. Chemotherapy seems to be important for HCC since it recovers individuals with an advanced grade, although the results have been confused [2, 3]. The unsatisfactory response could be explained by the non-targeted feature of the presently utilized drugs, as well as their poor delivery to cancer [3, 4]. Cancer nanotechnology is one of the technologies that we previously used in hepatic [5, 6], breast [7], and colorectal [8] cancer treatment. The development of cancer therapy has benefited greatly from the use of targeted-based drug delivery systems since they could deliver medications to the target locations with minimal dosing and in a controlled way to reduce adverse effects [9].

In our recent publication [6], we discovered new active drug-loaded nanoparticles conjugated with a selective moiety that could arrest the HCC cell cycle through binding with specific receptors that were highly expressed on the surface of the hepatoma cells. The cytotoxicity of this nanoformula was significant against HCC cells via regulation of the CDC25A/p53/PI3k pathway. This functional nanoformulation was synthesized using drug-loaded nanoparticles consolidated with aptamer on their surfaces.

Folded single-stranded nucleic acids (ssDNA or ssRNA) are known as aptamers (APT). They can bind to the target candidates with excellent affinity owing to their small molecular weights and lack of immunogenicity. Considering that they are intended to act as extracellular ligands for particular cell surface receptors, thus they are good candidates for targeted cancer therapy [10].

APT called AS1411 was explored to induce apoptosis in breast cancer cell lines [11]. AS1411 aptamer selectively tackles cancer cells because it attaches to nucleolin receptors. These receptors are overexpressed on the membranes of cancer cells thus, it has been successfully utilized to monitor glioma cells [12, 13]. Nucleolin is thought to be implicated in APT binding and engulfment processes, leading to the development of novel drug delivery strategies [14, 15]. As a result, we hypothesized that AS1411 aptamer may be utilized as a new tool for selective apoptosis of HCC cells.

Taribavirin or viramidine (VRM) was derived from a chemical modification of ribavirin, which is the old known hepatitis C viral therapy. VRM is known to be selective to the infected hepatic cells. Intriguingly, we recently employed nanotechnology focused on the possible use of VRM as an anti-cancer option against HCC and breast cancer cells [16, 17].

The main goal of this project was to investigate the apoptotic track of the targeted nanoparticle-based drug delivery system that can prevent cancer from progressing and evading chemotherapy due to the chemotherapy's non-selective nature. To achieve this goal we designed a selective AS1411 APT-labeled VRM-loaded NPs to introduce an active dual-targeted therapy for HCC in vitro.

## Methods

### Chemicals

Taribavirin, called viramidine (VRM), polyethylene glycol (PEG), propidium iodide (PI), 3-[4,5-dimethylthiazol-2-yl]-2,5-diphenyl tetrazolium bromide (MTT), N-hydroxysuccinimide (NHS), and N-(3-Dimethyl aminopropyl)-N’-ethyl-carbodiimide hydrochloride (EDC) were purchased from Sigma Aldrich (St. Louis, MO, USA). Dulbecco’s Modified Eagle’s Medium (DMEM), trypsin–EDTA, and penicillin–streptomycin (P/S), fetal bovine serum (FBS), and phosphate-buffered saline (PBS) were obtained from Life Technologies, Gibco (Grand Island, NY, USA). RNase-free water and all other reagents were of analytical grade and met the quality criteria in compliance with international principles.

### AS1411 APT preparation

The AS1411 DNA aptamer was purchased from (Aptagen, LLC, Jacobus, PA 17,407), with the sequence of 5'- GGT GGTGGTGGT TGT GGT GGTGGT GG -3'. The calculated molecular weight (Mwt.) of AS1411 APT was 8272.3 [6]. The AS1411 APT sequence was de-protected using ammonium hydroxide and methylamine at 65 °C for 30 min. The sequence was then further purified using reverse-phase HPLC (ProStar, USA) on a C-18 column with triethylamine acetate and acetonitrile. After being dried, the DNA product was detritylated by dissolving it and allowing it to sit in acetic acid for 20 min, then precipitated using ethanol and sodium chloride [6].

The proposed APT was examined using analytical electrospray ionization mass spectrometry (ESI–MS). Before being analyzed, ions were helped to move from the solution into the gaseous phase using electrical energy. Three processes are involved in the ESI transfer of ionic species from the solution into the gas phase: fine spraying of charge droplets, solvent evaporation, and ion ejection from the highly charged droplets, as previously mentioned [6].

#### Preparation of the nanoformulations

##### VRM NPs

It was prepared using an emulsion/solvent evaporation procedure as described previously [18, 19] after making some modifications. The emulsification was performed using a probe sonicator (Sonics & Materials Inc., USA) for 30 s at 80 W (F1) then increased to 120 W (F2). We found that F1 at 80 W recorded the best EE% and characterization properties, as discussed previously [6]. The synthesized VRM NPs were introduced to dialysis procedures against RNase-free water to remove the unencapsulated VRM.

##### *APT NPs*

AS1411 APT NPs were prepared through the formation of a covalent bond between PEG NP and the functional groups of the APT using the EDC/NHS technique according to the method reported before [6].

##### *APT-VRM NPs*

The labeling of AS1411 APT on the surface of the fabricated VRM NPs was carried out as described recently in our prior research [6] using an EDC/NHS technique according to the method of Cheng et al. [19].

##### Nano void (NV))

The nanoparticles without VRM or APT are called nanovoids (NV). These NPs were prepared as mentioned in detail in our recently published study [6].

#### Characterization of the nanoformulations

##### Particle size and zeta potential

Distribution of particles size and zeta potential of NV, VRM NPs, APT NPs, and APT-VRM NPs were determined by the Malvern Zeta sizer apparatus (Malvern Instruments, Westborough, Massachusetts), as described recently [6].

##### Entrapment efficiency (EE%)

The EE% of the VRM NPs and APT-VRM NPs was performed to determine the encapsulated VRM in the nanoparticles. The prepared NPs were introduced to a dialysis tube (Amicon 10,000 MWCO ultrafilter, Millipore, USA) for eliminating the impurities and the free non-entrapped VRM.

##### Transmission electron microscopy (TEM)

Particle's morphology was examined using TEM for the synthesized nanoparticles. Imaging software and digital micrograph were used to capture and analyze the images using Electron Microscope Unit at National Research Centre [6].

### FTIR and 1H NMR spectra

FTIR and ^1^H NMR spectra of APT-VRM NPs were determined using the same procedures mentioned recently [6].

### Cell lines

Human hepatocellular carcinoma HepG2 and Huh-7 cell lines, as well as BHK-21 normal cells were acquired from VACSERA and purchased from American Type Culture Collection (ATCC). These cells were propagated by adding DMEM medium completed with 10% FBS and 1% P/S. To endorse the proposed experiments, the cultured cells were first treated with 0.25% (w/v) trypsin/EDTA and then incubated in 5% CO_2_ at 37 °C.

#### SRB-based cytotoxicity and IC_50_ measurement

The cytotoxic effects of VRM, APT, APT + VRM, VRM NPs, APT NPs, and APT + VRM NPs as well as the standard anti-cancer drugs, doxorubicin (DOX), at the concentrations (0, 20, 40, 60, 80, 100 μM) were investigated on HepG2 and Huh-7 human liver cancer cell lines versus normal BHK-21 cell line using sulphorhodamine-B (SRB) assay according to Skehan's method [20]. Noting that the measuring unit of APT serial dosages was in nM. Briefly, the cells were seeded in 96 well plates at a concentration of 10,000 cells/well and left for surface attachment on the plate overnight in 5% CO_2_ at 37 °C. Then, cells were incubated for 48 h with the proposed treatments. After that, the cells were fixed with 10% trichloroacetic acid (TCA) for 1 h, then washed with distilled water 3 times. Cells were stained for 30 min at room temperature with 0.4% SRB dissolved in 1% acetic acid at room temperature. After incubation, the SRB solution was poured off and the plates were washed and the dye was solubilized with 150 μl/well of 10 mM tris base solution (PH 7.4). The optical density (OD) of each well was measured spectrophotometrically at A_540_ nm with an ELISA microplate reader. The experiment was performed in triplicate and the half maximal inhibitory concentration (IC_50_) values of HepG2, Huh-7, and BHK-21 cell viabilities were obtained using polynomial concentration–response curve fitting models (OriginPro 8 Software).

#### Apoptosis using flow cytometry technique

The flow cytometry method was utilized to detect the non-apoptotic and apoptotic populations of HepG2 and Huh-7 cells as well as early and late apoptotic cell distributions upon the proposed treatments. One million HCC cancerous cells were seeded and left for 24 h period. These cancerous cells were incubated for 48 h with the IC_50_ dosages. The used IC_50_ values were obtained from the MTT-based cytotoxicity performed in very recent publication [6], which also confirmed in the current study using SRB-based cytotoxicity (please see Table 1). After incubation with the treatments, all cells were stained with Annexin V and PI dyes. By using a Beckman flow cytometer (USA), the apoptosis of the treated and untreated (control) cells was examined.

**Table 1:** SRB-based cytotoxicity of HepG2, Huh-7, and BHK-21 cell lines

Cells/ IC_50_	VRM	APT	APT + VRM	VRM NPs	APT NPs	APT + VRM NPs	DOX
HepG2 cell line	43.07 ± 0.73	42.91 ± 3.20	38.61 ± 1.75	27.69 ± 1.35	31.09 ± 0.65	16.69 ± 1.12	88.56 ± 3.95
Huh-7 cell line	44.06 ± 2.50	50.21 ± 1.34	36.90 ± 2.35	20.13 ± 0.88	19.62 ± 0.57	11.23 ± 0.23	90.68 ± 0.71
BHK-21 cell line	90.43 ± 4.50	95.21 ± 3.25	94.15 ± 2.23	ND	ND	ND	86.25 ± 3.46

IC_50_; the drug dosage at which 50% of the cells were killed. Units of the measured IC_50_; µM for VRM and DOX, while nM for APT. *ND* Not-detectable (means IC_50_ recorded more than 100 µM). HepG2 and Huh-7 cells; human hepatic cancerous cell lines. BHK-21 cell line; normal cell line

#### Genetic expression of the apoptotic pathway

Total RNA was isolated from both HCC cell lines after 48 h of the therapeutic application using the RNeasy mini kit (Qiagen, Germany) according to the manufacturer’s guidelines. As mentioned before, the concentrations of the used formulas against HepG2 and Huh-7 cells were as reported in (Table 1). According to the operating instructions, the cDNA was produced using a cDNA synthesis kit. We bought kits and primers from Thermo Fisher Scientific (USA). Quantitative expression levels of FADD**,** c-FLIP**,** caspase 8, caspase 3, caspase 9, Bax, Bcl-2, and TNF-α genes were carried out. The utilized primers' sequences were tabulated in Table 2. The housekeeping gene was GAPDH. To count the copies of the cDNA, a real-time PCR cycler (DT lite 4, Russia) was used. A mixture of 25 µL from SYBR Green, primers, RNase-free water, and cDNA was used to start up PCR reactions. The reaction program was designated as the following. The first step was 3 min at 95.0 °C. The second phase was made up of 35 cycles, each of which was broken into three steps: (a) 15 s at 95 °C; (b) 30 s up to 60 °C; and (c) 30 s at 72 °C. At the end of each qRT-PCR, a melting curve analysis was constructed to check the quality of the utilized primers.

**Table 2:** Primer sequences of the studied genes for real time qRT-PCR

Gene	Primer sequence	
FADD	F	5’- CTCAGGTCCTGCCAGATGAAC-3’
	R	5’-GGACGCTTCGGAGGTAGATG-3’
c-FLIP	F	5'-ATGTCTGCTGAAGTCAT CC-3'
	R	5'-ATCCTCACCAATCTCCTGCC-3'
Caspase8	F	5’- AGAGTCTGTGCCCAAATCAAC-3’
	R	5’- GCTGCTTCTCTCTTTGCTGAA- 3’
Caspase3	F	5'GTGGAACTGACGATGATATGGC3'
	R	5'CGCAAAGTGACTGGATGAACC3'
Caspase9	F	5′-AACCCTAGAAAACCTTACCCC-3′
	R	5′-CATCACCAAATCCTCCAGAAC-3′
Bax	F	5'-ATGGCTTCTATGAGGCTGAG-3'
	R	5'-CGGCCCCAGTTGAAGTTG-3'
Bcl-2	F	5'-CTGCACCTGACGCCCTTCACC-3'
	R	5'-CACATGACCCCACCGAACTCAAAGA-3'
TNF-A	F	5'-CTGAACTTCGGGGTGATCG-3'
	R	5'-GCTTGGTGGTTTGCTACGAC-3'
GAPDH	F	5'-GTCTCCTCTGACTTCAACAGCG-3'
	R	5'-ACCACCCTGTTGCTGTAGCCAA-3'

F = forward primer

R = reverse primer

#### Protein levels of the apoptotic pathway

The protein levels of caspase 8, caspase 3, caspase 9, Bax, Bcl-2, and TNF-α were assessed after treating human HCC cancerous cells with the proposed formulations for 48 h using their IC_50_ dosages by enzyme-linked immunosorbent assay (ELISA) kits obtained from Sunlong Biotech Co. (China). These ELISA kits use Sandwich-ELISA as the method. The Microelisa strip plates provided in these kits were pre-coated with the antibody specific to each target protein. Standards of each protein or sample were added to the appropriate Microelisa strip plate wells and combined with the specific antibody. Then Horseradish Peroxidase (HRP)-conjugated antibody specific for each target protein was added to each well in the used plate. Free components were washed away and the specific substrate solution was added to each well. Only wells that contain the target protein and HRP conjugated protein antibody showed a colored appearance. The change of color, after the addition of the stop solution, was measured spectrophotometrically at a specific wavelength to each target protein. Each OD value is proportional to the concentration of the corresponding target protein. The concentration of each target protein in the samples was calculated by comparing the OD of the samples to the standard curve.

#### Stress markers

##### Nitric oxide (NO)

Nitric oxide was measured by colorimetric kits purchased from (Spectrum Diagnostics, Egypt). Briefly, in 96-well plates, the cells were grown at a density of 1 × 10^4^ cells per well. The proposed IC_50_ doses of the different prepared formulations mentioned in (Table 1) were added to the culture media on the second day. First, nitrate reductase was used to change nitrate into nitrite. Then, nitrite was transformed into an intensely purple azo molecule using Griess reagent. The concentration of NO in the samples was properly reflected by the amount of azo chromophore. And finally, the microplate reader was used to detect optical density at A_540_ nm (BMG Labtech, Germany).

##### Inducible nitric oxide synthase (iNOS)

Inducible nitric oxide synthase enzyme activity was quantified after treatment of human HCC cancerous cells with the tested formulations using an ELISA kit purchased from (Wuhan Fine Biotech Co., China) according to the manufacturer's manual. The quantitative sandwich enzyme immunoassay method was used in this assay. A microplate was pre-coated with an iNOS-specific antibody. The enzyme was bound by the immobilized antibody after pipetting standards or samples into the wells. A biotin-conjugated antibody specific for iNOS was added to the wells after removing any unbound compounds. Avidin-conjugated Horseradish Peroxidase (HRP) was then added to the wells after washing. A substrate solution was subsequently added to the wells after washing to remove any unbound avidin-enzyme reagent. The developed color was proportionally to the amount of iNOS bound in the initial phase. The color development was paused and the microplate reader was used to measure the color's intensity at A_540_ nm (BMG Labtech, Germany).

### Statistical analysis

One-way ANOVA was used for comparison between the treated groups and the control group. *; means significant difference (*P* <0.05) and #; means high significant difference (*P* <0.01) compared to control. Results were expressed in the term of mean ± standard error (SE).

## Results

### Characterization of the synthesized nanoparticles

#### Transmission electron microscopy (TEM)

As illustrated in our prior work [6], TEM showed spherical morphology of VRM NPs with smooth surfaces, and the spherical balls of these nanoparticles had the size of nano-scale. APT-VRM NPs clarified by TEM revealed a spherical shape with two partitions (partition 1: the core of the NPs containing the loaded VRM; partition 2: the labeled chains of the AS1411 aptamer). The composition of the APT-VRM NPs led to greater sphere size than VRM NPs as discussed by our prior study [6].

#### Particle size, polydispersity, Zeta potential, and EE%

After optimization of the NPs, the average size of VRM NPs was in nano grade. About double increase in size was observed following its conjugation with APT (APT-VRM NPs). The zeta potential of VRM NPs was shown to be negatively charged and the labeling with APT significantly reduced the zeta potential of APT NPs and APT-VRM NPs as illustrated recently [6]. The results of EE% of the optimized VRM NPs and VRM-APT NPs were also explained in our previously published research [6] indicating the best EE% values.

### Characterization of APT NPs

According to our most recent report [6], FTIR and ^1^H NMR validated the chemical structure of the PEG NPs and the created link in the produced APT NPs.

### Measurement of cytotoxic and apoptotic signatures in HCC cell lines

#### *SRB and IC*_*50*_* output*

The effects of VRM, APT, APT + VRM, VRM NPs, APT NPs, APT + VRM NPs, and DOX on HepG2, Huh-7, and BHK-21 cell proliferations were monitored using SRB assay. The SRB dye is responsible for binding with cellular amino acids/ proteins, and its colorimetric estimation provides an evaluation of total protein in the detected cells. It was noticed that the proliferation of the proposed HCC cells were significantly suppressed in a concentration and formulation-dependent manner recording different detectable IC_50_ values, while the normal BHK-21 cells were slightly affected upon using free drugs and recording undetectable IC_50_ values upon using nano-formulations (VRM NPs, APT NPs, APT + VRM NPs) (Table 1). IC_50_, which was identified as the concentration of the used formula at which 50% of cancer/normal cells are died, suggested that Huh-7 cells recorded the lowest IC_50_ value (11.23 ± 0.23 µM) compared to HepG2 cells (16.69 ± 1.12 µM) upon APT + VRM NPs treatment. Followed by APT NPs application which noted 19.62 ± 0.57 µM and 31.09 ± 0.65 µM for Huh-7 and HepG2 cells, respectively. The IC_50_ values recorded by VRM NPs application on Huh-7 and HepG2 cells were relatively near to the results of APT NPs (Table 1).

Regarding free drugs, the combination between APT and VRM provides enhanced cytotoxic effects and lower IC_50_ results (Huh-7 cells: 36.90 ± 2.35 µM and HepG2 cells: 38.61 ± 1.75 µM) than individual APT drug (Huh-7 cells: 50.21 ± 1.34 µM and HepG2 cells: 42.91 ± 3.2 µM) as well as VRM drug (Huh-7 cells: 44.06 ± 2.5 µM and HepG2 cells: 43.07 ± 0.73 µM). The results of normal BHK-21 cells approved the effective targetability of the synthesized nanoparticles, where the IC_50_ values for free drugs and DOX were very high (around 90 µM) compared to cancerous cells, while the IC_50_ values for nano-drugs were undetectable (Table 1). Noting that, high IC_50_ values means minimal cytotoxic effect on normal cells.

#### Apoptosis

Recently, we studied the cytotoxicity and cell cycle phases of APT-VRM NPs on HCC cells, and herein we tracked the apoptotic pathways using VRM, APT, APT-VRM, NV, VRM NPs, APT NPs, and APT-VRM NPs in the two proposed HCC cell lines (HepG2 and Huh-7). The tools used for that purpose include flow cytometric analyses and genetic expression of the extrinsic apoptotic (FADD**,** caspase 8, caspase 3, and caspase 9), intrinsic or mitochondrial apoptotic (Bax and Bcl-2), and survival (TNF-α) genes.

### Flow cytometric analyses

**Fig. 1:**
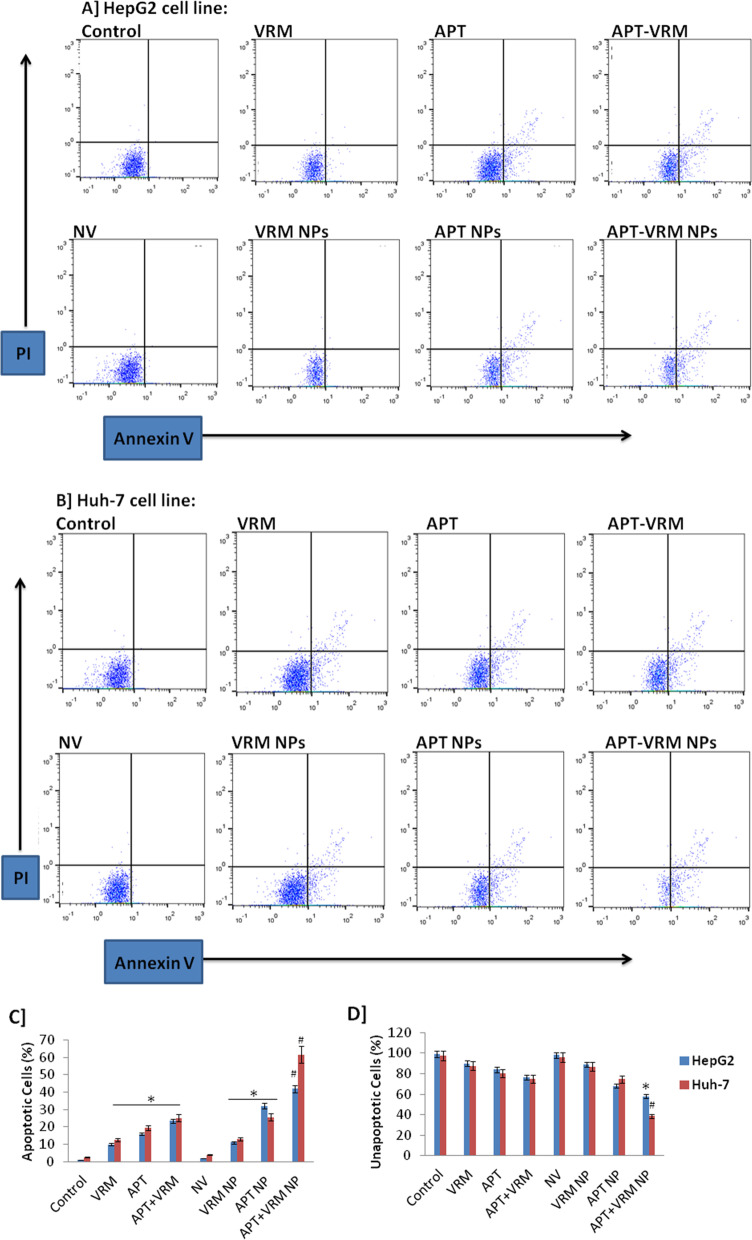
Apoptosis using flow cytometry analyses. **A** Representative figures of apoptosis using HepG2 cells. **B** Representative figures of apoptosis using Huh-7 cells. **C** Graphical representation of apoptotic HepG2 and Huh-7 cells (*n* = 3). **D** Graphical representation of the nonapoptotic (healthy population) in HepG2 and in Huh-7 cells. *; means significant difference (*P* <0.05) and #; means high significant difference (*P* <0.01) compared to control

Flow cytometry investigation offers the ability to study large numbers of cells individually rather than in a mixed population. Figure 1A, B represented the apoptotic diagrams of HepG2 and Huh-7 cells, while Fig. 1C, D illustrated the column bars in the term of means and SE.

#### Nonapoptotic population

It was evident that the count percent of HepG2 cells in the quadrant of negative annexin V/negative PI (nonapoptotic cells) showed a gradual decrease after VRM, APT or APT-VRM treatments, recording 90%, 84%, and 76.5%, respectively. Regarding the treatment with the nanoformulations, the count percent of HepG2 cells in the quadrant of negative annexin V/negative PI (nonapoptotic cells) revealed a significant decrease in a gradual manner after VRM NPs, APT NPs, or APT-VRM NPs application, recording 89%, 68%, and 58%, respectively, registering the highest significant decrease in the percent of the nonapoptotic cells with APT-VRM NPs (58%).

Concerning the count percent of Huh-7 cells in the quadrant of negative annexin V/negative PI (nonapoptotic cells), it exhibited a gradual decrease but more than that of HepG2 cells upon VRM, APT or APT-VRM treatments, recording 87.5%, 80.5%, and 74.8%, respectively. For treatment with nanoformulations, the count percent of Huh-7 cells in the quadrant of negative annexin V/negative PI (nonapoptotic cells) was significantly decreased gradually (more than that of HepG2 cells) after VRM NPs, APT NPs, or APT-VRM NPs handling, recording 87%, 74.5%, and 38.5%, respectively, registering the highest significant reduction in the percent of the nonapoptotic cells with APT-VRM NPs (38.5%).

#### Apoptotic population

The count percent of HepG2 cells in the three quadrants: (1) positive annexin V/negative PI (early apoptotic cells), (2) positive annexin V/positive PI (late apoptotic cells), and (3) negative annexin V/ positive PI (necrotic cells) showed gradual increase upon treatment with VRM, APT or APT-VRM, recording 10%, 16%, and 23.5%, respectively. In regard to the treatment with the nanoformulations, the count percent of HepG2 cells in the three mentioned quadrants revealed a significant increase in a gradual pattern upon VRM NPs, APT NPs, or APT-VRM NPs treatment, recording 11%, 32%, and 42%, respectively, registering the highest significant increase in the percent of apoptotic cells with APT-VRM NPs (42%).

In respect to the count percent of Huh-7 cells in the three quadrants: (1) positive annexin V/negative PI (early apoptotic cells), (2) positive annexin V/positive PI (late apoptotic cells), and (3) negative annexin V/ positive PI (necrotic cells), it displayed gradual increase after VRM, APT or APT-VRM treatment, recording 12.5%, 19.5%, and 25%, respectively. Upon treatment with the nanoformulations, the count percent of Huh-7 cells in the three mentioned quadrants showed a gradual significant increase (more than that of HepG2 cells) regarding VRM NPs, APT NPs, or APT-VRM NPs application, recording 13%, 25.5%, and 61.5%, respectively, registering the highest significant increase in the percent of the apoptotic cells with APT-VRM NPs (61.5%).

### Genetic expression of FADD and c-FLIP

**Fig. 2:**
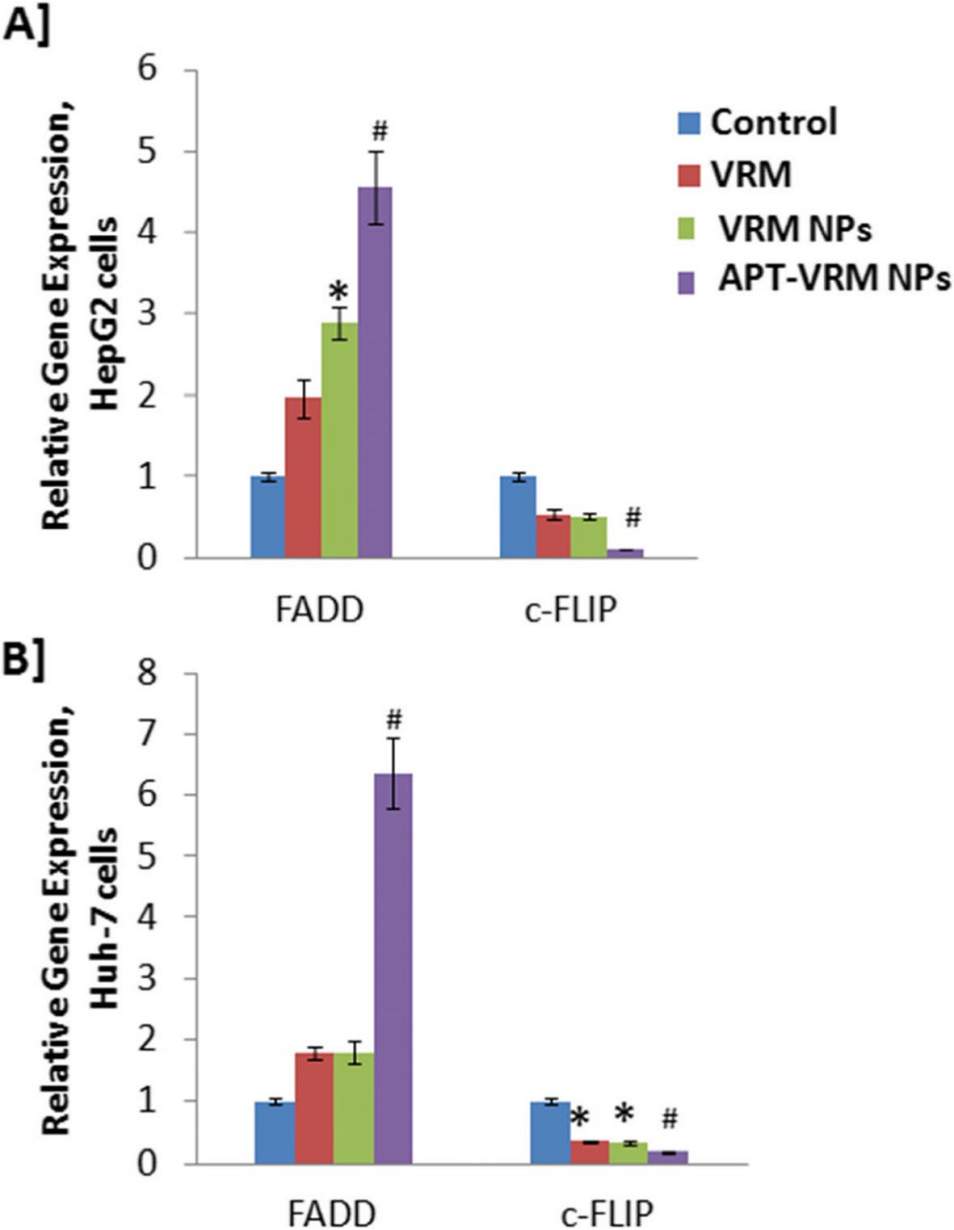
Genetic expression levels of FADD and c-FLIP. **A** Genetic expression levels of of FADD and c-FLIP in HepG2 cells. **B** Genetic expression levels of of FADD and c-FLIP in Huh-7 cells (*n* = 3). *; means significant difference (*P* <0.05) and #; means high significant difference (*P* <0.01) compared to control

The expression of FADD and c-FLIP genes in human hepatic HepG2 and Huh-7 cancerous cell lines was investigated (Fig. 2). Treatment of HepG2 and Huh-7 cells with the IC_50_ dose of VRM, VRM NPs, or APT-VRM NPs significantly up-regulated FADD and down-regulated c-FLIP genes expression by contrast with the control, in both cell lines, with a greater effect on Huh-7 cells. HepG2 cancerous cells treated with the IC_50_ dose of APT-VRM NPs recorded highly significant up-regulation (*P* < 0.01) in the FADD gene expression (4.5 fold increase) and highly significant down-regulation (*P*<0.01) in the c-FLIP gene expression (0.11 fold decrease) compared to the control (1), as shown in Fig. 2A. Huh-7 cancerous cells treated with the IC_50_ dose of APT-VRM NPs recorded highly significant up-regulation (*P* <0.01) in FADD gene expression (6.35 fold increase) and highly significant down-regulation (*P* <0.05) in the c-FLIP gene expression (0.18 fold decrease) versus the control (1), as represented in Fig. 2A.

### Genetic expression and protein levels of caspases (8, 3, and 9)

**Fig. 3:**
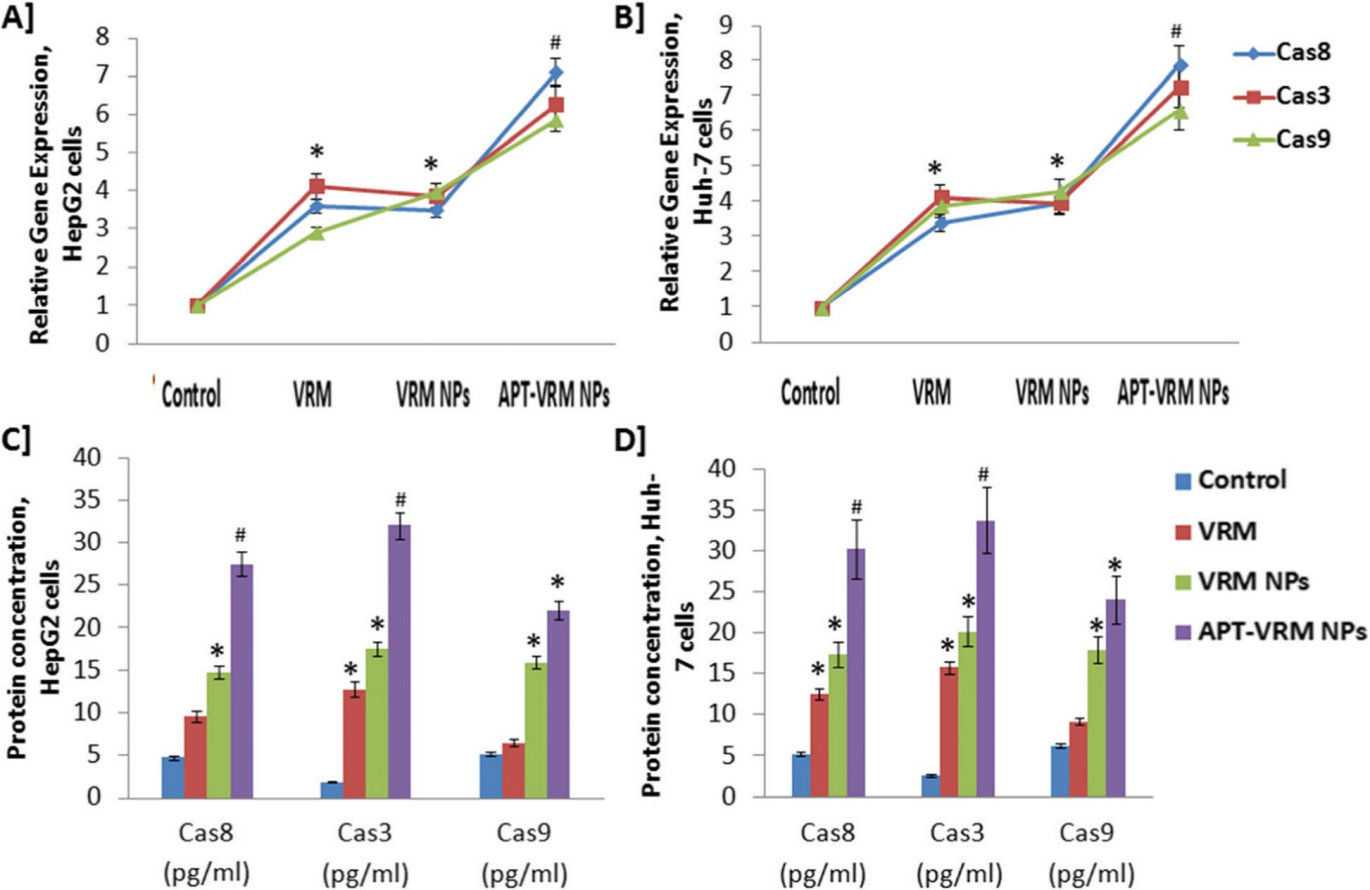
Genetic expression and protein levels of caspases (8, 3, and 9). **A** Genetic expression levels of caspases (8, 3, and 9) in HepG2 cells. **B** Genetic expression levels of caspases (8, 3, and 9) in Huh-7 cells (*n* = 3). **C** Protein concentrations of of caspases (8, 3, and 9) in HepG2 cells. **D** Protein concentrations of caspases (8, 3, and 9) in Huh-7 cells (*n* = 3). *; means significant difference (*P* <0.05) and #; means high significant difference (*P* <0.01) compared to control

The gene expression and protein levels of caspases (8, 3, and 9) in human hepatic HepG2 and Huh-7 cancerous cell lines were studied (Fig. 3A, B respectively). Treatment of HepG2 and Huh-7 cells with the IC_50_ dose of VRM, VRM NPs, or APT-VRM NPs significantly up-regulated (*P* < 0.05) caspases (8, 3, and 9) genes expression contrary to the control in both cell lines, with a greater impact on Huh-7 cells. HepG2 cancerous cells treated with the IC_50_ dose of APT-VRM NPs recorded highly significant up-regulation (*P* <0.01) in the caspases (8, 3, and 9) genes expression (7.1, 6.27, and 5.86 fold increase, respectively) in comparison with the control (1) as illustrated in Fig. 3A. Huh-7 cancerous cells treated with the IC_50_ dose of APT-VRM NPs registered the highest significant up-regulation (*P* <0.01) in the caspases (8, 3, and 9) genes expression (7.91, 7.27, and 6.6 fold increase) comparative to the control (1) as manifested in Fig. 3B. Protein level of caspases (8, 3, and 9) showed a similar increasing pattern of their genetic expression (Fig. 3C, D).

### NO level and iNOS enzyme activity

**Fig. 4:**
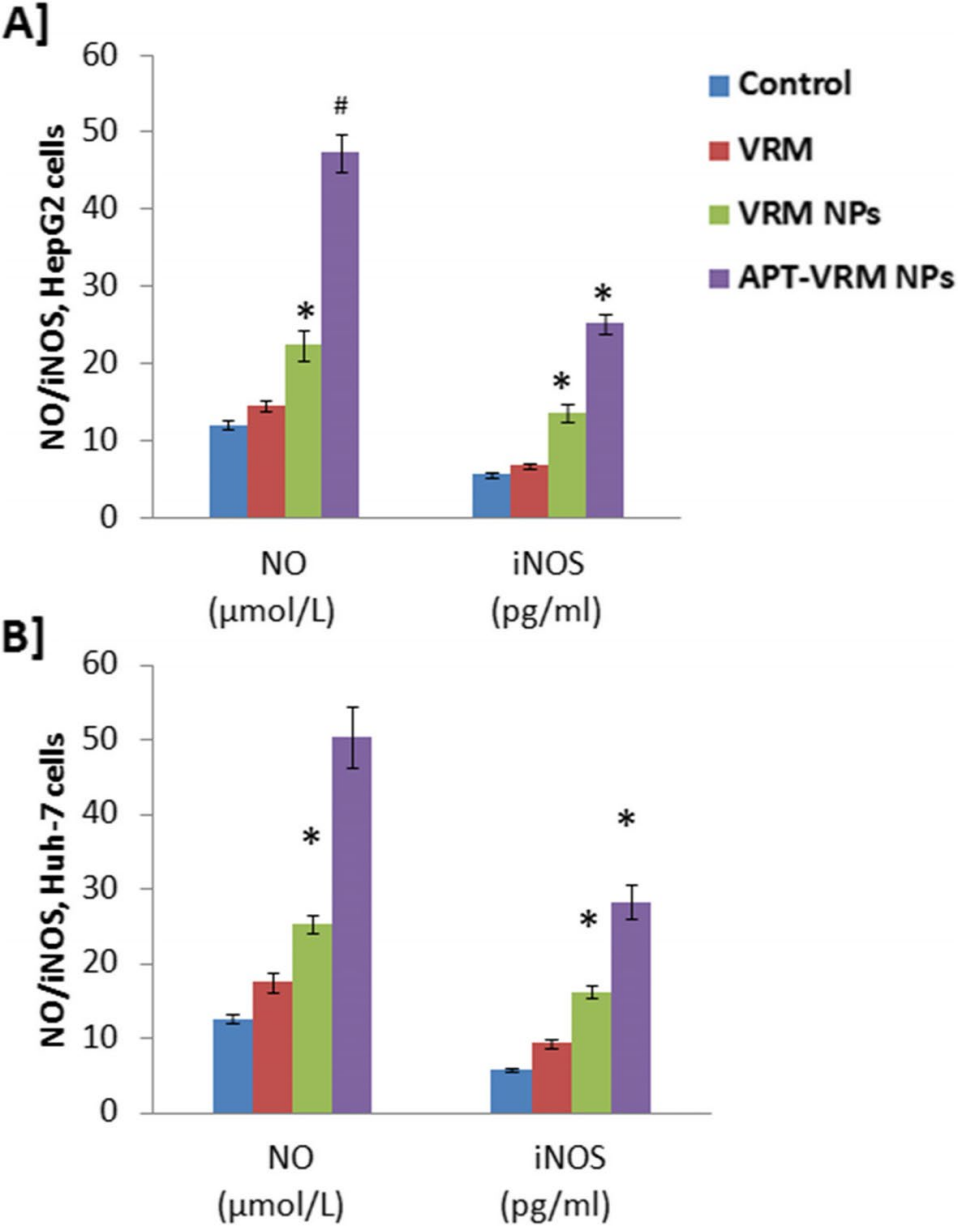
APT-VRM NPs elevate NO through activation of iNOS. **A** NO and iNOS levels in HepG2 cells. **B** NO and iNOS levels in Huh-7 cells (*n* = 3). *; means significant difference (*P* <0.05) and #; means high significant difference (*P* <0.01) compared to control

Nitric oxide (NO) levels, as a reactive nitrogen species (RNS), and the activity of its synthesized enzyme (iNOS) were measured in human hepatic HepG2 and Huh-7 cancerous cell lines (Fig. 4A, B respectively). Treatment of HepG2 and Huh-7 cells with the IC_50_ dose of VRM, VRM NPs, or APT-VRM NPs significantly elevated (*P* < 0.05) NO level and iNOS activity compared to the control in both cell lines, with a greater influence on Huh-7 cells. APT-VRM NPs elevated NO levels through the activation of iNOS which stimulates apoptosis.

### Genetic expression and protein levels of Bax, Bcl-2, and TNF- α

**Fig. 5:**
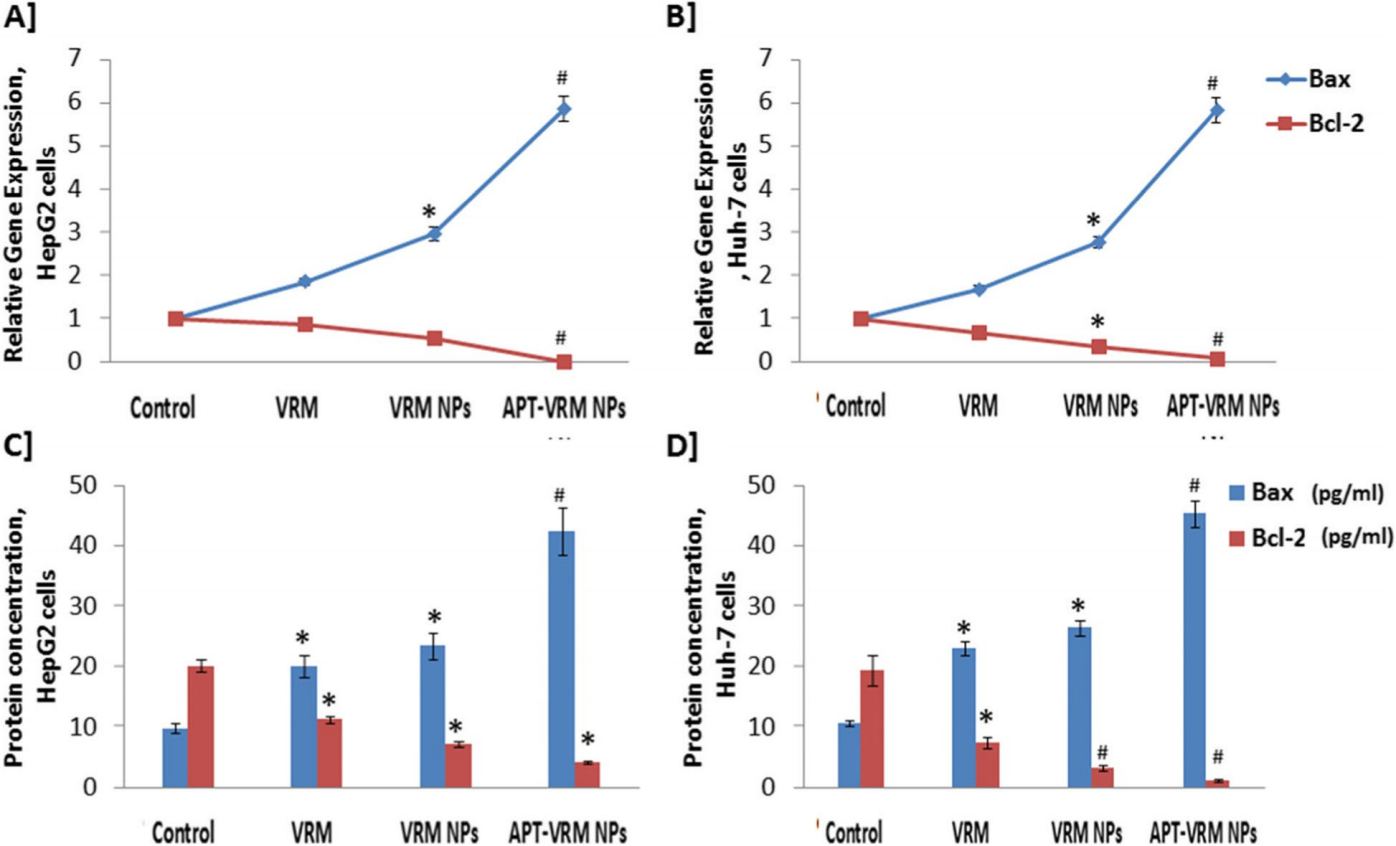
Genetic expression and protein levels of Bax and Bcl-2. **A** Genetic expression levels of Bax and Bcl-2 in HepG2 cells. **B** Genetic expression levels of Bax and Bcl-2 in Huh-7 cells (*n* = 3). **C** Protein concentrations of Bax and Bcl-2 in HepG2 cells. D] Protein concentrations of Bax and Bcl-2 in Huh-7 cells (*n* = 3). *; means significant difference (*P* <0.05) and #; means high significant difference (*P* <0.01) compared to control

**Fig. 6:**
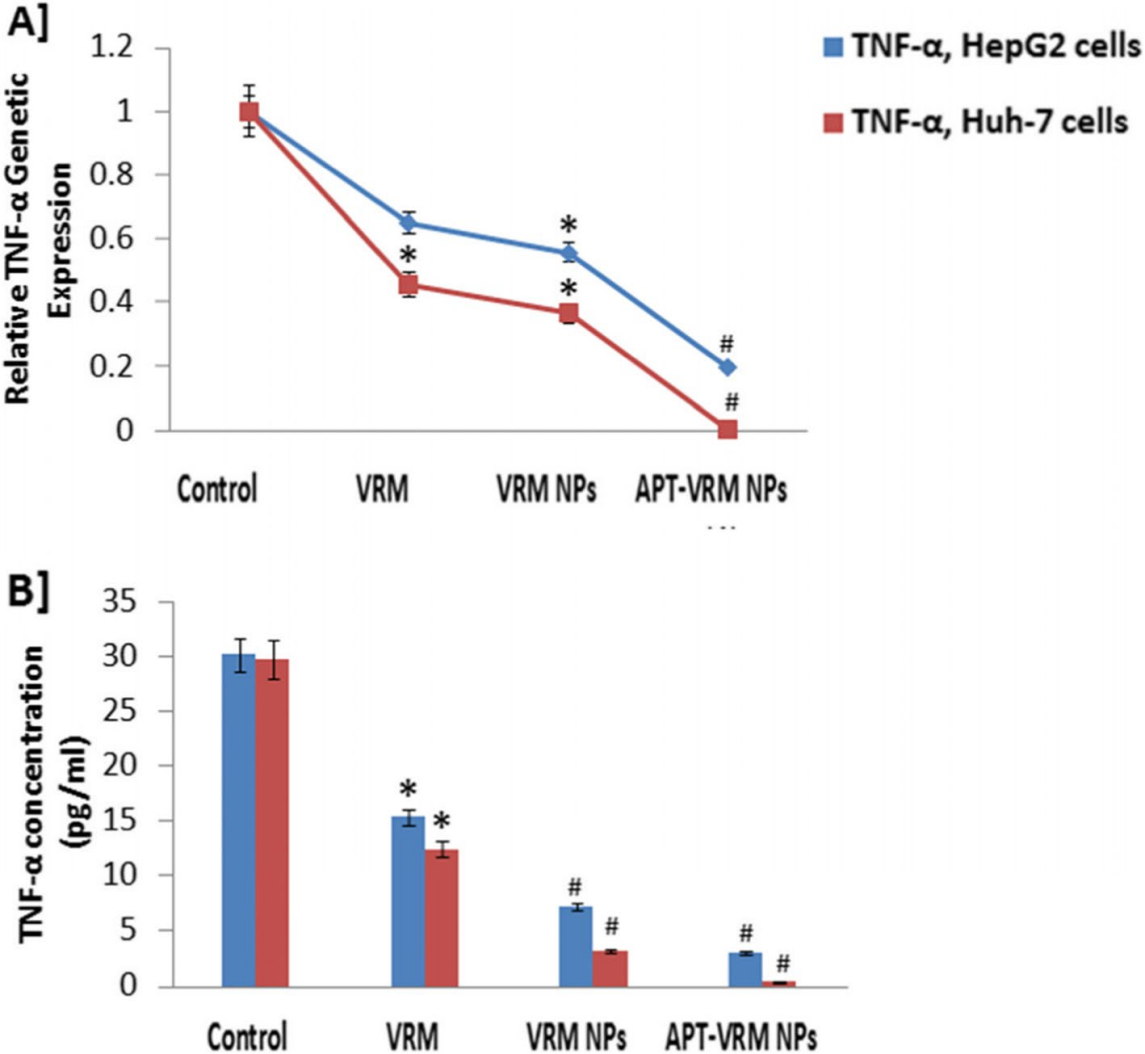
TNF-α genetic expression and protein levels. **A** TNF- α genetic expression levels in HepG2 and Huh-7 cells. **B** A] TNF- α protein concentrations in HepG2 and Huh-7 cells (*n* = 3). *; means significant difference (*P* <0.05) and #; means high significant difference (*P* <0.01) compared to control

The expression of Bax, Bcl-2, and TNF- α genes and their related protein levels in human hepatic HepG2 and Huh-7 cancerous cell lines were examined (Figs. 5 and 6). Treatment of HepG2 and Huh-7 cells with the IC_50_ dose of VRM, VRM NPs, or APT-VRM NPs significantly up-regulated Bax and down-regulated Bcl-2 and TNF- α (*P* < 0.05) gene expression. Off note, the protein levels of these genes behaved in the same pattern versus the control in both cell lines, with a greater effect on Huh-7 cells. HepG2 cancerous cells treated with the IC_50_ dose of APT-VRM NPs recorded highly significant up-regulation (*P* <0.01) in Bax gene expression (5.85 fold increase) and significant down-regulation (*P* <0.05) in Bcl-2 and TNF-α genes expression (0.01 and 0.2 fold decrease, respectively) contrary to control (1) as demonstrated in Figs. 5 and 6A. Huh-7 cancerous cells treated with the IC_50_ dose of APT-VRM NPs registered highly significant up-regulation (*P* <0.01) in Bax gene expression (5.82 fold increase) and highly significant down-regulation (*P* <0.01) in Bcl-2 and TNF-α genes expression (0.081 and 0.008 fold decrease, respectively) by contrast with the control (1) as evidenced in Figs. 5 and 6A.

Table 3 elucidated the Bax/Bcl-2 ratio in HepG2 and Huh-7 cells upon VRM, VRM NPs, and APT-VRM NPs treatments. APT-VRM NPs recorded the highest Bax/Bcl-2 ratio in HepG2 and Huh-7 cancerous cell lines (58.5 and 71.24, respectively) versus the control (1), noting that Huh-7 cells showed the greatest Bax/Bcl-2 ratio upon treatment with APT-VRM NPs compared to other treatments. The protein level of Bax, Bcl-2, and TNF-α in Figs. 5 and 6B displayed a similar pattern as their genetic expression.

**Table 3:** Bax/Bcl-2 ratio in HepG2 and Huh-7 cell lines

Cells vs. Bax/Bcl-2 ratio	Control	VRM	VRM NPs	APT-VRM NPs
HepG2 cells	1	2.12	5.28	58.5
Huh-7 cells	1	2.48	7.51	71.24

## Discussion

In our recently published manuscript, we studied the cell cycle and its relevant genes in HepG2 and Huh-7 cell lines after the application of the proposed formulations including VRM, APT, APT-VRM, NV, VRM NPs, APT NPs or APT-VRM NPs [6]. Herein, we discussed an impressing mechanistic track (extrinsic and intrinsic apoptotic pathways) in HCC cell lines upon treatment with the same formulations.

The synthesized nanoparticles showed high selectivity in addition to their suitable nano-size required for drug delivery, allowing the loading NPs to pass through the membrane spaces in cancer cells while avoiding crossing through the normal cells' spaces, particularly APT-VRM NPs which had nano-size of less than 150 nm [6].

DNA-based aptamers like AS1411, C-2, TLS11a, and Bio-TLS11a [21–23] have been generated against human hepatic HepG2 malignant cell lines. When compared to nearby non-cancerous cells, they were selectively bound to malignant sites. In this attempt, we chose AS1411 DNA APT because of its high staining in the most abundant site of nucleolin in hepatic cancerous cells [23].

Nucleolin is overexpressed in many actively dividing cancerous cell lines and absent or downregulated in normal cells therefore nucleolin has been widely employed as a targeted receptor. It was suggested that the quantity of AS1411 internalized by cancerous cells associates with the nucleolin expression level regardless of cell type [9]. Recently, we measured nucleolin expression levels and monitored the binding affinity in HepG2 and Huh-7 cell lines using APT and VRM NPs [6]. We also studied cytotoxicity of both cancerous cell lines upon different treatments using mitochondrial-dependant assay (MTT). Significant improvement in cellular association between nucleolin and APTNP was observed in Huh-7 cell line higher than HepG2 cell line, thus an increased MTT-based cytotoxic effect against Huh-7 cell line over 48 h was seen [6]. Herein, in the current study, we performed the cytotoxicity experiment using another protein-dependant assay (SRB) and added a third normal cell line (BHK-21) to test the targetability of our synthesized nanoparticles against cancerous cell lines (HepG2 and Huh-7). We found that all used formulas reported relatively similar IC_50_ results against HepG2 and Huh-7 cell lines regardless of the used method type either based on mitochondrial (MTT) or protein (SRB) staining. Again, it was suggested that Huh-7 cells had the lowest IC_50_ value (11.23 µM) using SRB assay and (13.99 µM) using MTT assay upon 48 h incubation of APT-VRM NPs, suggesting that the protein-based SRB assay noted slight higher cytotoxic increase compared to mitochondrial-based MTT assay. Intriguigely, the undetectable IC_50_ readings for the nano-formulations in case of normal BHK-21 cells confirming the active and safe targeting power of the proposed NPs against cancerous cells, while sparing normal cells with minimal cytotoxic effect.

The focus of our interest in the current study on the extrinsic and intrinsic (mitochondrial) apoptotic pathways, upon treatment with the synthesized candidate (APT-VRM NPs), was due to the good knowledge that gained from our previous publications, in which VRM nanoparticles were succeeded to deliver VRM as an anti-cancer agent against HCC [16] and breast [17] cancerous cells. In addition to VRM, it was established that aptamer AS1411 DNA induces apoptosis in breast cancer cell lines [11]. Besides, the induction of apoptosis, AS1411 APT showed high selectivity to cancer cells while sparing normal cells, owing to its ability to bind receptors that are highly expressed on the cancer cell membrane called nucleolin [6, 13, 14].

Apoptosis is a healthy cell death type in which a cell dies as a result of a sequence of molecular occurrences. This is one method the body uses to get rid of unneeded or abnormal cells. This process is blocked in cancer cells, thus common defiance in developing cancer therapy is the induction of cancer cell apoptosis. The extrinsic pathway of this process can be triggered through the ligand-receptor specific connection on the membranes of the cancerous cells, while the intrinsic or mitochondrial pathway of apoptosis can be stimulated by intracellular signals [24]. In regards to ligand-induced apoptosis in the present study, APT is considered as a ligand and the corresponding death receptor is nucleolin. Our recent study showed the binding affinity between APT and nucleolin and how that connection induced cytotoxicity [6]. Herein, from the obtained results, we may suggest that APT NPs induce HCC cell death through extrinsic apoptosis, as our findings showed the induction of extrinsic apoptotic genes such as FADD**,** caspase 8, caspase 3, and caspase 9.

Additionally, the gathered results in the current investigation revealed that cell death by APT-VRM NPs may be mediated by early and late apoptosis along with minor contribution of necrosis was noticed (i.e. negative annexin V and positive PI). However, extraordinary late apoptosis conquers at the IC_50_ dose of the APT-VRM NPs. These findings are consistent with recent research showing that VRM-loaded NPs cause apoptosis in breast and liver cancer cells [16, 17]. It was stated that free VRM also produces necrosis in addition to late apoptosis in non-healthy cancer cells. VRM NPs elicit both early and late apoptosis. When compared to control, VRM NPs collectively boosted the apoptosis index further, indicating that the NPs enhance the early apoptotic impact of VRM by facilitating its entry into the cells [16].

The underlying mechanism by which APT-VRM NPs caused apoptosis in HepG2 and Huh-7 cells was the activation of the APT-receptor link, which could provide apoptotic signals via their intracellular death domain (DD) [25], sequenced by recruitment of death domain (FADD) [26]. FADD was up-regulated in liver cancerous cell lines employing NPs in the current investigation, with the highest percentage in APT-VRM NPs against Huh-7 cells (6.35%) and HepG2 cells (4.5%) compared to control (1). The main complex (DISC) is formed when the FADD adaptor protein recruits pro-caspase 8. When pro-caspase 8 is recruited, it activates and then cleaves a cascade of subsequent caspases, causing membrane blebbing, DNA breakage, and nuclear shrinkage [26]. Thus, in the current study, caspase 8, caspase 3, and caspase 9 were up-regulated upon APT-VRM NPs treatment in HCC cells, especially Huh-7 cells which showed more sensitivity than HepG2 cells.

In some cases, activated caspase 8 needs a mitochondrial response in what is called the intrinsic apoptotic route. Bax, Bcl-2-associated X protein, attaches to tBid and then translocates to the mitochondria. The polarization of the mitochondrial membrane then changes [26] and tBid also causes mitochondrial cytochrome c release [27], resulting in the formation of an apoptosome structure. This apoptosome activates caspase 9 and other caspases [28].

Bax and Bcl-2 are two important members of the Bcl-2 family that play a key role in regulating mitochondrial apoptosis. This family is divided into anti-apoptotic (Bcl-2) and pro-apoptotic (Bax) [29, 30]. Bcl-2 up-regulation and Bax down-regulation have both been linked to cancer resistance [31, 32]. The ratio of Bax to Bcl-2 appears to play an important function in modulating cancer cell apoptosis susceptibility. Shifting this balance toward apoptosis makes it a feasible strategy for killing cancer cells [33]. As a result, in the current approach, when HCC cells were treated with APT-VRM NPs, Bax was down-regulated and Bcl-2 was up-regulated, especially in Huh-7 cells, which had the highest Bax/Bcl-2 ratio (71.24) compared to the control (1).

Although the activation of pro-caspase 8 through DISC formation is considered a key step in the onset of the extrinsic apoptotic signal, an inhibitory protein nominated c-FLIP similar to caspase 8, may block the activation of caspase through direct competition on FADD. Some TNF receptors called (TRAF2 and TRADD) are important in forming a secondary complex in the presence of c-FLIP [34]. Thus, in the present work, APT-VRM NPs treatment reduced the expression of c-FLIP and TNF- α in HCC cells, particularly Huh-7 cells, where c-FLIP and TNF- α were found to record the greatest inhibition values (0.18 and 0.008, respectively) compared to control (1). On the contrary, it was reported that c-FLIP can act as a pro-apoptotic candidate [35].

Reactive nitrogen species (RNS), such as NO and its enzyme iNOS, may be a source of DNA damage and activation of p53 leading to stimulation of extrinsic track of apoptosis through FADD and caspases up-regulation [36], or activation of intrinsic apoptotic track through Bax up-regulation [37]. In addition, the activators of the extrinsic apoptosis may act by NF-kB downregulation and other survival tracks [34]. Thus, in the current study, NO levels and iNOS activity were elevated upon APT-VRM NPs treatment in HCC cells, especially Huh-7 cells, in which the NO and iNOS achieved the highest values compared to control and other treatments.

## Conclusion

To sum up, the current study provides scientific evidence that AS1411 APT functionalization on VRM nanoparticle surfaces (APT-VRM NPs) has promising pro-apoptotic activity in hepatocellular carcinoma (HepG2 and Huh-7) cell lines. The behind mechanism is mediated by NO elevation that is stimulated by the activation of iNOS enzyme. The synthesized nanoformulation actively manipulated both extrinsic and intrinsic apoptosis through up-regulation of the extrinsic apoptotic genes (FADD, caspase 8, caspase 3, and caspase 9) and enhancement of the Bax/Bcl-2 ratio (intrinsic or mitochondrial apoptotic genes), and inhibition of survival TNF-α gene.

## Data Availability

The datasets used and/or analyzed during the current study are available from the corresponding author upon reasonable request.
